# Genetic mapping of the *p47 ^L.3.2^* mutation in *Drosophila* *melanogaster*

**DOI:** 10.17912/micropub.biology.000783

**Published:** 2023-09-18

**Authors:** Chloe N Cordes, Melissa M. Gouge, Hannah Morgan, Carah Porter, Jamie Siders, Erica Bacab, Briana Barin, ixel A. Becerra, Jose Castillo, Jared Church, Shaunacee Condo-Hicks, Kattleya J Conroy, Isbel Curbelo-Navarro, Benjamin J. DePrest, Adrienne Eady, TyMahni Edmead, Verena Farouk, Angelica Flores-Torres, Michael Girmai, Cynthia I. Gutierrez Navarro, Stan Guzman, Alexis B Harris, Karin Healy, Alina Jaafar, Abraham Khadr, Melissa N Kiboi, Stephanie N Korte, Celia Lopez, Hyder Mahdi, Jennifer Mendoza Avalos, Kathy Miranda, Dipti Patel, Celia Lopez, Hyder Mahdi, Jennifer Mendoza Avalos, Kathy Miranda, Dipti Patel, Rishi Patel, Sydney Pechulis, Victoria Plachta, Kamryn Rhodes, Andrea M Sandoval, Caila Thomas, Joselyn A. Valadez-Mendoza, Hannah Vora, Jonathan Yousif, Kayla L Bieser, Jacob D Kagey

**Affiliations:** 1 Ohio Northern University, Ada, OH, USA; 2 Nevada State University, Henderson, NV, USA; 3 University of Detroit Mercy, Detroit, MI, USA

## Abstract

An EMS-based forward genetic screen was conducted in an apoptotic null background to identify genetic aberrations that contribute to regulation of cell growth in *Drosophila melanogaster* . The current work maps the genomic location of one of the identified mutants, *L.3.2* . Genetic crosses conducted through the Fly-CURE consortium determined that the gene locus for the *L.3.2* mutation is *p47* on chromosome 2R.

**Figure 1.**
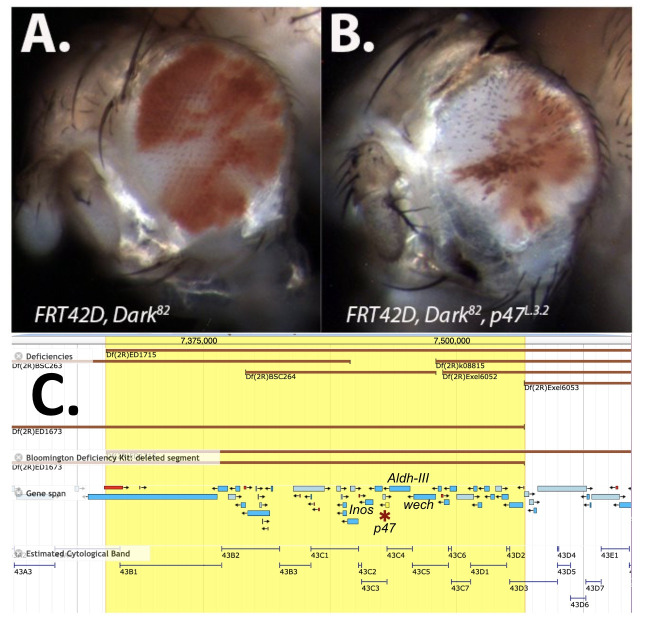
**
The *Dark ^82^ , p47 ^L.3.2^* mutant results in abnormal cellular structure and hair growth:** A.) Lateral view of *FRT42D, Dark ^82^* mosaic control eye. Eye tissue from control mosaic *(FRT42D, Dark ^82^* ) display a slight R>W phenotype as previously identified (Akdemir et al, 2006). The *Dark* mosaic eyes have a regular eye shape and size. B.) Lateral view of eyes from mosaic *FRT42D, Dark ^82^ ,p47 ^L.3.2^ . Dark ^82^ , p47 ^L.3.2^* mosaic eyes displayed W>R phenotypes. These also depicted a strong rough eye phenotype and overall reduction in eye size. All eye images captured at 40x. C.) A screenshot of the JBrowse genome browser accessed through FlyBase.org, highlighting the region that failed to complement, via crosses to Df(2R)ED1715 (BDSC 8931) and Df(2R)ED1673 (BDSC 9062); 2R:7,395,885.. 7,489,834. Subsequent single allele complementation mapping demonstrated that the allele, *p47 ^L.3.2^* (Larkin, 2021) failed to complement (red asterisk).

## Description

An ethyl methanesulfonate (EMS) based genetic screen was conducted in *Drosophila* aimed at identifying regulators of cell growth, cell division, and development. EMS mutations were induced in *FRT42D, Dark ^82^* flies, which are null for *Dark* activity ( *Dark ^82^* ) and possess a mw ^+^ cassette. Conducting the screen in a *Dark* null mosaic background blocks apoptosis, facilitating identification of a broader range of genetic contributors to cell growth and development (Akdemir et al, 2006, Kagey et al., 2012) . Mutagenized flies ( *FRT42D,Dark ^82^ ,L.3.2* ) were crossed to *FRT42D; Ey-Flp* flies to yield mosaic clones of homozygosity in eye tissue. Control *FRT42D,Dark ^82^ x FRT42D; Ey-Flp* crosses result in eyes with a fairly normal developmental patterning and a slight increase in the ratio of red:white pigmentation to about 60:40 ( Figure 1A ). Progeny that were mosaic for *FRT42D,Dark ^82^ ,L.3.2* yielded progeny with a rough eye pattern depicting disrupted ommatidial structure and greater white:red pigmentation, shifting from the *Dark ^82^* mosaic control ( Figure 1B ).

In order to identify the gene locus responsible for the rough phenotype observed in the L.3.2 mosaic flies, *FRT42D,Dark ^82^ ,L.3.2* virgin female flies were crossed in serial to males from the Bloomington Stock Center 2R deficiency kit (Cook et. al., 2012) . Complementation crosses were conducted by undergraduate students at the following Fly-CURE consortium institutions: University of Detroit Mercy, Nevada State University, and Ohio Northern University. From these crosses, Df(2R)ED1715 (BDSC 8931) and Df(2R)ED1673 (BDSC 9062) failed to complement, narrowing the gene region to 2R:7,395,885.. 7,489,834 ( Fig. 1C, Table 1). Single gene mutants of alleles from this narrowed down region were then crossed to *FRT42D,Dark ^82^ ,L.3.2* flies. Crosses between *Mi{MIC}p47 ^MI13219^ /SM6a* (BDSC 58033) x *FRT42D,Dark ^82^ ,L.3.2* failed to complement, while crosses to three adjacent genes, *Inos (P{SUPor-P}Inos ^KG07679^ ]/CyO* (BDSC14921)), *Aldh* ( *P{EPgy2}Aldh-III ^EY12056^ /CyO* (BDSC 20339), and *wech* ( *P{lacW}wech ^k08815^ /Cy* O (BDSC 10818)), resulted in complementation (Table 1). This data confirms L.3.2 as a novel lethal allele of *p47* , *p47 ^L.3.2^* .

Based on the genetic data presented, we determined that the *L.3.2* phenotype genetically mapped to the gene *p4* 7. This *p47 ^L.3.2^* allele is located at 2R:7,465,018..7,466,759 ( Figure 1C ). p47 is known to regulate the ATPase activity of p97, a member of the AAA+ superfamily of ATPases (Meyer et. al., 1998) . Molecular interactions between p97 and p47 are implicated in a variety of cellular processes, including ER and golgi membrane fusion (Kondo et. al., 1997; Uchiyama and Kondo, 2005) , mitotic spindle disassembly (Cao et. al., 2003) nuclear envelope formation (Hetzer et. al., 2001) , and autophagosome biogenesis (Krick et. al., 2010) . Evidence for a role of p47 in regulating cell growth comes from studies of the human p47 ortholog, NSFL1C. Studies in the MCF10AT breast cancer model show that NSFL1C is a target of epidermal growth factor signaling (Chen et. al., 2007) , while recent work in a human adult T-cell leukemia/lymphoma (ATLL) cell line demonstrates that inhibition of autophagy-mediated p47 degradation ultimately leads to restoration of caspase-3 mediated apoptosis and decreased rates of cell growth (Fauzi et. al., 2021) . The connection between loss of p47 and apoptosis induction, may provide insight as to why p47 was missed in the initial round of Flp/FRT screens, and suggests p47 may be a conditional growth suppressor. Finally, the rough eye phenotype observed in the *p47 ^L.3.2^* mutant flies agrees with early work on *p47* in flies demonstrating that a gain-of-function mutation in *p47* disrupts rhodopsin processing, suggesting a role for *p47* in proper eye development (Sang and Ready, 2002) . Further functional characterization of *p47* protein activity in *Drosophila* will be required to delineate its role in eye development.

**Table 1** . Complementation results with BDSC 2R deficiency lines. Additional deficiency lines not found in the 2R kit and individual alleles crossed to *FRT42D, Dark ^82^ , L.3.2* flies are also displayed *.* Deficiency mapping narrowed down the region of the *L.3.2* mutation to 2R:7,395,885.. 7,489,834, which contains the gene *p47* . Crosses of *FRT42D,Dark ^82^ ,L.3.2* x y ^1^ w*;Mi{MIC}p47 ^MI13219^ /SM6a failed to complement, establishing *p47* as the location of the L.3.2 mutation.

**Table:** 

**Bloomington 2R Deficiency Kit**			
**Deficiency**	**BDSC Stock #**	**Region**	**Complementation Result**
*Df (2R)ED1715*	8931	2R:7,326,951..7,916,923	Fail to Complement
*Df (2R)ED1725*	8941	2R:7,613,924..8,156,045	Fail to Complement
*Df (2R)ED1673*	9062	2R:6,985,802..7,533,553	Fail to Complement
**Additional Deficiency Lines**			
**Deficiency**	**BDSC Stock #**	**Region**	Complementation Result*
*Df (2R)BSC264*	23163	2R:7,395,885..7,489,834	Fail to Complement
*Df (2R)BSC263*	23162	2R:7,146,864.. 7,447,410	Complement
**Single Genes Tested Within Fail to Complement Region**			
**Gene**	**BDSC Stock #**	**Allele**	**Complementation Result**
*p47*	58033	Mi{MIC}p47 ^MI13219^ , *2R:7464671..7467108*	Fail to Complement
*Inos*	14921	P{SUPor-P}Inos ^KG07679^ *, 2R:7454134..7459668*	Complement
*Wech*	10818	P{lacW}wech ^k08815^ *, 2R:7476659..7492017*	Complement
*Aldh-III*	20339	P{EPgy2}Aldh-III ^EY12056^ *, 2R:7464829..7479226*	Complement
* The Fly-CURE consortium employs the following guidelines for determination of genetic complementation indicated in this table. 1. Crosses designated as 'Complement' resulted in at least 10 straight wing progeny when scored. 2. Crosses designated as 'Fail to Complement', resulted in at least 100 curly wing progeny and 0 straight wing progeny when scored.			

## Reagents

*w ^-^ ; FRT42D, Dark ^82^ /CyO* (Akdemir et al., 2006)

*w ^-^ ; FRT42D Dark ^82^ , p47 ^L.3.2^ /CyO* (this study)

*w ^-^ ; FRT42D; Ey-Flp* (BDSC 8211)

Bloomington *Drosophila* Stock Center 2R Deficiency Kit *(Cook et al., 2012)*

*Additional Bloomington Stocks (See Table 1 for complete list of stock numbers)*
